# Cord serum brain-derived neurotrophic factor levels at birth associate with temperament outcomes at one year

**DOI:** 10.1016/j.jpsychires.2022.03.009

**Published:** 2022-06

**Authors:** Hayley Dingsdale, Samantha M. Garay, Hannah R. Tyson, Katrina A. Savory, Lorna A. Sumption, Jemima S. Kelleher, Kate Langley, Stephanie Van Goozen, Rosalind M. John

**Affiliations:** a School of Biosciences, Cardiff University, Cardiff, CF10 3AX, UK; b School of Psychology, Cardiff University, Cardiff, CF10 3AT, UK; c Cardiff University Centre for Human Developmental Science, School of Psychology, Cardiff University, Cardiff, CF10 3AT, UK

**Keywords:** Attention, Brain-derived neurotrophic factor, Neurodevelopment, Temperament

## Abstract

Altered serum levels of brain-derived neurotrophic factor (BDNF) are consistently linked with neurological disorders. BDNF is also increasingly implicated in the pathogenesis of neurodevelopmental disorders, particularly those found more frequently in males. At birth, male infants naturally have significantly lower serum BDNF levels (∼10–20% lower than females), which may render them more vulnerable to neurodevelopmental disorders. We previously characterized serum BDNF levels in mothers and their newborn infants as part of the Grown in Wales Study. Here, we analyzed whether cord serum BDNF levels at birth correlate with sex-specific outcomes at one year. The Bayley Scale of Infant Development, Third Edition (BSID-III) and Laboratory Temperament Assessment Battery (Lab-TAB) tasks were used to assess infant behavior and neurodevelopment at 12–14 months (mean ± SD: 13.3 ± 1.6 months; 46% male; n = 56). We found no relationship between serum BDNF levels at birth and BSID-III neurodevelopmental outcomes (cognitive or language), nor with infant behaviors in the Lab-TAB unpredictable mechanical toy or maternal separation tasks. In the sustained attention task, there was a significant positive relationship between serum BDNF and infant negative affect (*B* = 0.06, *p* = 0.018) and, for boys only, between serum BDNF and intensity of facial interest (*B* = 0.03, *p* = 0.005). However, only the latter remained after correction for multiple testing. This sex-specific association between cord serum BDNF and a parameter of attention at 12–14 months provides some support for the hypothesis that reduced serum BDNF levels at birth are linked to an increased risk for neurodevelopmental disorders.

## Highlights

• BDNF is implicated in neurodevelopmental disorders more frequently seen in males
• Male infants have lower serum BDNF at birth; this may factor into higher risk
• Serum BDNF at birth was not linked to cognitive or language outcomes at 1 year
• In males only, serum BDNF positively associated with facial interest in attention
• Serum BDNF at birth may associate with risks for neurodevelopmental disorders

## 1 Introduction

Brain-derived neurotrophic factor (BDNF) is critical to the healthy development and function of the brain (Park and Poo, 2013), and dysregulated levels of the neurotrophin are found in numerous neurological disorders (Lima Giacobbo et al., 2019). Lower serum BDNF levels have been reported in patients with depression (Brunoni et al., 2008; Karege et al., 2002a; Kishi et al., 2018; Molendijk et al., 2014; Polyakova et al., 2015; Sen et al., 2008), and neurodegenerative disorders including Alzheimer's disease (Ng et al., 2019) and Huntington's disease (Ciammola et al., 2007). Conversely, higher levels of circulating BDNF have been shown in children and adults with autism (Barbosa et al., 2020; Liu et al., 2021; Qin et al., 2016). BDNF in the blood is synthesized by megakaryocytes (Chacon-Fernandez et al., 2016) and present in circulating platelets (Rosenfeld et al., 1995). Blood BDNF has been hypothesized to reflect levels found in the brain (Klein et al., 2011). This is supported by the findings of a strong correlation between levels of cortical and serum BDNF in rats, particularly in the early postnatal period (Karege et al., 2002b). Serum BDNF levels are known to increase with gestational age (Cai et al., 2017; Cannon et al., 2008; Chouthai et al., 2003; Flock et al., 2016) and it has been hypothesized that levels of serum BDNF may be positively associated with neural maturity (Chouthai et al., 2003). These observations suggest that measures of blood BDNF may provide both a useful biomarker of neurological disease and insight into the pathogenesis of neurodevelopmental disorders.

Male infants naturally have serum BDNF levels approximately 10–20% lower than females at birth (Chouthai et al., 2003; Dingsdale et al., 2021; Flock et al., 2016; Spulber et al., 2010; Yu et al., 2021). Consequently, these naturally lower levels of BDNF in boys early in life could render male infants at increased risk of neurodevelopmental disorders. Attention-deficit hyperactivity disorder (ADHD) is of key interest in this context, as multiple lines of evidence already link BDNF and ADHD (Tsai, 2003). Animal models of this disorder show dysregulated BDNF expression (Fumagalli et al., 2003), while conversely ablation of BDNF in animal models produces hyperactivity (Kernie et al., 2000; Rios et al., 2001). Pharmacologically, BDNF levels are altered centrally and peripherally by two different ADHD medications (methylphenidate and atomoxetine) (Akay et al., 2018; Amiri et al., 2013; Fumagalli et al., 2010). It is not yet clear whether blood BDNF levels are significantly different in people with ADHD, with conflicting reports published (see Supplementary Table 1) (Akay et al., 2018; Allred et al., 2017; Amiri et al., 2013; Bilgic et al., 2017; Chang et al., 2020; Corominas-Roso et al., 2013; Cubero-Millan et al., 2017; Lee et al., 2015, 2017; Li et al., 2014; Mansur et al., 2016; Ramos-Quiroga et al., 2014; Sahin et al., 2014; Scassellati et al., 2014; Seyedi et al., 2019; Shim et al., 2008; Simsek et al., 2016; Skogstrand et al., 2019; Vogel et al., 2017; Wang et al., 2019; Yeom et al., 2016; Yurteri et al., 2019; Zeni et al., 2016).

Previously, we characterized serum BDNF levels in mothers (n = 251) and their newborn infants (n = 212) as part of the larger Grown in Wales (GiW) Study (Dingsdale et al., 2021). These infants were then followed up at 12–14 months, and the Bayley Scale of Infant Development, Third Edition (BSID-III) and Laboratory Temperament Assessment Battery (Lab-TAB) were used to assess infant development and temperament (Savory et al., 2020). The BSID-III is often used to identify children at risk of developmental delay and can assess multiple domains, including cognitive, language and motor (Del Rosario et al., 2021). Lab-TAB tasks are an objective method of assessing infant behaviors and temperament. It is a useful alternative to parentally-completed questionnaires, although the association between parent- and lab-reported behaviors is modest (Planalp et al., 2017). Infant temperament is relatively stable, with certain dimensions of infant temperament predicting increased risks of adverse outcomes much later in childhood, including anxiety behaviors (Kagan, 2002) and ADHD traits (Goodwin et al., 2021).

Here, we analyzed the relationship between infant serum BDNF levels at birth and these infant outcomes, to identify whether cord serum BDNF levels associate with later temperament and neurodevelopmental measures.

## 2 Methods

### 2.1 Participants

Women were originally recruited as part of the GiW pregnancy cohort study. Inclusion criteria were: singleton term pregnancy in women aged between 18 and 45, with no fetal abnormalities or infectious diseases. Recruitment took place at pre-surgical appointments prior to elective caesarean section (ELCS) at the University Hospital of Wales. Initially 355 women gave written informed consent after the procedures were fully explained; 7 later withdrew from the study. From these mother-infant dyads, serum BDNF measurements were achieved for 212 infant samples. Infant serum BDNF was measured from cord blood taken within 2 h of delivery, processed as described previously (Dingsdale et al., 2021). Briefly, blood was collected into Vacutainer blood tubes, inverted, and incubated for 1–2 h at room temperature (RT), before centrifugation at 3000×*g* for 10 min, 4 °C. Serum was then collected and stored at −80 °C prior to measurement.

All births were at 37 weeks or over. Criteria used to exclude samples from the dataset prior to analysis were based on previous studies and included non-white mothers and infants (n = 1) (Christian et al., 2016), and infant samples when mothers went into natural labor prior to their planned ELCS (n = 2) (Flock et al., 2016).

### 2.2 Serum BDNF measurements

Infant serum BDNF levels reported in this study were previously reported in the context of maternal mood disorders at birth (Dingsdale et al., 2021). Serum BDNF levels were measured using a previously validated ELISA ((Naegelin et al., 2018); with minor modifications), based on the publicly available BDNF antibodies, Ab#1 and Ab#9 (Kolbeck et al., 1999). In brief, after 3 washes with Buffer A (0.1% Triton X-100 in 0.1 M phosphate buffer: 0.1 M KH_2_PO_4_, 0.1 M Na_2_HPO_4_, pH7.6), NeutrAvidin-coated plates (ThermoFisher Scientific, 15509) were incubated for 2 h at RT with 13 μg/ml biotin-conjugated Ab#1 in Buffer A. Plates were then washed with Buffer B (Buffer A with 1% BSA (Sigma, A2143)), prior to 6 h incubation at RT with either samples or standards (recombinant BDNF; Regeneron/Amgen) diluted in Buffer B. Samples and standards were measured in triplicate wells, and plates contained 2 repeated samples to monitor consistency between ELISAs. After this incubation step, wells were washed with Buffer A, prior to a further 3 h RT incubation with HRP-conjugated Ab-#9 at 1.25 μg/ml in Buffer B. Finally, wells were washed with Buffer A and chemiluminescent substrate (Roche, 11582950001) added, with signal detected by microplate reader (FLUOstar OMEGA, BMG Labtech). ELISA measurements were performed blind to all information except sample type (maternal serum or cord blood serum) and participant ID.

### 2.3 Infant laboratory assessment

When infants reached approximately 12 months of age, mother-infant dyads were invited to participate in a lab-based assessment (Savory et al., 2020). Eighty-three dyads returned, for which cord serum BDNF levels were available for fifty-seven infants. Mothers provided written informed consent for this stage of the study after the procedures were fully explained; all assessments and analysis were performed blind to serum BDNF measurements. The BSID-III was used to assess age-standardized language and cognitive development. Scores were analyzed as either continuous variables, or categorized as “at risk”, “emerging”, or “competent” using cut-off scores as defined by the Bayley's manual and equipment.

Several tasks from the Lab-TAB were used to assess various parameters of infant temperament (Goldsmith and Rothbart, 1996). Here we report the unpredictable mechanical toy task, the sustained attention task, and the maternal separation task, performed as previously described (Savory et al., 2020). In the unpredictable mechanical toy task, a novel robotic toy was used to assess fear. Infants were seated for the task, with mothers instructed to avoid interacting with their child. For each of 3 trials, the robot was walked toward the child and stopped approximately 20 cm away. Here, it was paused for 10 s and then returned to its starting place, before a further 5 s pause. Infants were given the opportunity to interact with the robot at the end of the third trial. If it was necessary to end the task prematurely, coding was continued with the score in the last eligible epoch. For the maternal separation task, mothers were instructed to leave the room in their usual manner, leaving the infant in the room with just 1 experimenter for 2 min. During this time the infant had free access to play with any toy in the room. For the sustained attention task, infants were again seated. A carousel was placed approximately 40 cm from the infant and set to play for 3 min. Where the task had to be ended prematurely, coding was continued with the score in the last eligible epoch. Videos were excluded from analysis if medical issues preventing completion of the tasks were present (n = 1).

### 2.4 Statistical analysis

Data analysis was performed using RStudio V1.4.1103 (RStudio Team, 2021), and packages “dplyr” (Wickham et al., 2021), “flextable” (Gohel, 2021a), “ggplot” (Wickham, 2016), “gtsummary” (Sjoberg et al., 2021), “labelled” (Larmarange, 2021), “officer” (Gohel, 2021b), and “pastecs” (Grosjean and Ibanez, 2018). Data were assessed for normality using the Shapiro-Wilk test. As serum BDNF levels were not normally distributed, non-parametric analyses were performed where appropriate, including the Kruskal-Wallis test for group differences. Regression analysis was used to assess the relationship between cord serum BDNF levels and infant outcomes. Given the known effect of sex on cord serum BDNF levels (Chouthai et al., 2003; Dingsdale et al., 2021; Flock et al., 2016; Spulber et al., 2010; Yu et al., 2021), analyses were performed on both combined groups and split by sex. Age in months at the assessment was controlled for as a confounding variable, with parity also included in the supplementary analysis. Multiple testing was corrected for using the Benjamini-Hochberg Procedure, with each assessment (and Lab-TAB task) treated as a separate analysis.

### 2.5 Study approval

This research was performed in accordance with the Declaration of Helsinki as revised in 2008. The Grown in Wales Study was given full ethical approval by the Wales Research Ethics Committee (REC reference 15/WA/0004).

## 3 Results

Demographic data for the mother-infant dyads returning for the infant assessment at 12–14 months were previously reported (Savory et al., 2020). The cohort demographics, further restricted to dyads for whom infant cord serum BDNF measurements were available, is provided again here with the addition of data on BDNF serum levels (Table 1). Mothers returning for the Year 1 assessment were significantly older than non-returners (median: 35 vs. 33 years; *p* = 0.001) with higher Welsh Index of Multiple Deprivation scores (median: 1544 (within the 5th quintile/least deprived in the population) vs. 1270 (4th quintile); *p* = 0.030), indicative of areas with lower levels of deprivation. There was no difference in the infant serum BDNF levels between those returning for lab-based assessments vs. those not undertaking these assessments (*p* = 0.49). We previously reported significantly lower levels of serum BDNF from the cord blood of male infants in the original cohort (n = 212 (Dingsdale et al., 2021)). This significant difference in cord blood serum BDNF was retained in the subset of infants returning for assessment at 12–14 months (median (IQR); female infants: 10.17 ng/ml (8.28, 11.53) vs. male infants: 8.02 ng/ml (5.53, 9.46); *p* = 0.019 by Wilcoxon rank sum exact test).

**Table 1:** Demographics of mother-infant dyads.

	Non-returners (n = 155)	Y1 Attendees (n = 56)	*p*
**Maternal Age at birth (years), Median (IQR)**	33.0 (29.0, 36.0)	35.0 (32.0, 38.0)	**0.001**
**Parity, n (%)**			0.90
Nulliparous	32 (21%)	12 (21%)	
Multiparous	123 (79%)	44 (79%)	
**Infant Sex, n (%)**			0.94
Female	84 (54%)	30 (54%)	
Male	71 (46%)	26 (46%)	
**Education Level, n (%)**			**0.002**
Left before GCSE	14 (9.4%)	0 (0%)	
GCSE/Vocational	36 (24%)	5 (9.3%)	
A levels	19 (13%)	5 (9.3%)	
University	44 (30%)	20 (37%)	
Postgraduate	36 (24%)	24 (44%)	
Unknown	6	2	
**Family Income, n (%)**			**0.002**
<18,000	14 (9.5%)	0 (0%)	
18–25,000	15 (10%)	1 (1.8%)	
25–43,000	33 (22%)	8 (14%)	
>43,000	68 (46%)	40 (71%)	
Do not wish to say	18 (12%)	7 (12%)	
Unknown	7	0	
**WIMD Score, Median (IQR)**	1270 (407, 1685)	1544 (880, 1738)	**0.030**
Unknown	8	3	
**Infant cord serum BDNF (ng/ml), Median (IQR)**	9.57 (7.33, 11.94)	9.20 (7.27, 11.18)	0.49

Table 1: Only dyads for whom infant cord serum BDNF levels were available are included. *IQR*: Interquartile range; *WIMD*: Welsh Index of Multiple Deprivation. Low scores indicate areas of high deprivation. Bold values indicate *p* < 0.05.

Regression analyses were used to assess the relationship between infant serum BDNF levels at birth and BSID-III scores, controlling for infant age at assessment. None of the BSID-III parameters – cognitive, receptive language, and expressive language – showed a significant relationship with infant serum BDNF levels at birth (Table 2). There were also no significant differences in cord BDNF levels when infants were categorized as “at risk”, “emerging”, or “competent” for each of the three parameters (Kruskal-Wallis rank sum test: *p* = 0.49, 0.37, and 0.37 for cognitive, and receptive and expressive language scores, respectively). Given the lower levels of serum BDNF in cord blood from male infants, we also performed the same analysis in male and female infants separately; again no relationship between any of the BSID-III parameters measured and cord serum BDNF levels were identified (Table 2).

**Table 2:** Relationship between Infant Serum BDNF levels at birth and BSID-III cognitive and language outcomes at 12–14 months.

	All			Male			Female		
	*B*	95% CI	*p*	*B*	95% CI	*p*	*B*	95% CI	*p*
**BSID-III**									
Expressive language	0.12	−0.07, 0.31	0.204	0.04	−0.24, 0.33	0.755	0.13	−0.16, 0.43	0.354
Receptive language	0.04	−0.11, 0.20	0.574	−0.03	−0.27, 0.20	0.762	0.10	−0.13, 0.33	0.369
Cognitive	−0.02	−0.19, 0.15	0.836	-0.01	−0.19, 0.17	0.932	0.04	−0.26, 0.35	0.768

Controlled for Age at Assessment. Expressive language: n = 42 infants (20 male, 22 female); Receptive language: n = 44 infants (22 male, 22 female); Cognitive: n = 56 infants (26 male, 30 female). Bold values indicate *p* < 0.05. BSID-III: Bayley Scales of Infant Development Third Edition; CI: Confidence Intervals.

We next looked at relationships between cord serum BDNF levels at birth and infant behaviors in the Lab-TAB tasks. In regression models controlling for infant age at assessment, there was no relationship between cord serum BDNF levels at birth and infant fear-based behaviors in either the unpredictable mechanical toy task or the maternal separation task (Table 3). In the sustained attention task, several relationships were identified. There were significant relationships between cord serum BDNF levels and both parent behavior (*B* = 0.07, *p* = 0.009) and infant negative affect measures (*B* = 0.06, *p* = 0.018) (Table 3). Higher levels of serum BDNF were associated with a greater degree of parental interference during the task, and higher infant negative affect scores. These relationships remained when controlling for parity as an additional confounding variable (Table S2), but did not remain when correcting for multiple testing (correcting for 7 tests).

**Table 3:** Relationship between Infant Serum BDNF levels at birth and Infant behavior in three Lab-TAB tasks.

	All			Male			Female		
	*B*	95% CI	*p*	*B*	95% CI	*p*	*B*	95% CI	*p*
**Unpredictable mechanical toy (fear)**									
Facial fear	−0.07	−0.17, 0.03	0.168	−0.03	−0.19, 0.13	0.680	−0.11	−0.26, 0.05	0.164
Distress	−0.08	−0.20, 0.03	0.154	−0.08	−0.23, 0.07	0.272	−0.12	−0.32, 0.08	0.218
Bodily fear	0.00	−0.04, 0.04	0.965	0.01	−0.07, 0.09	0.744	0.00	−0.06, 0.05	0.859
Intensity of escape	−0.01	−0.05, 0.04	0.750	0.02	−0.03, 0.06	0.530	−0.03	−0.10, 0.05	0.438
Startle response	0.00	0.00, 0.00	0.100	0.00	0.00, 0.00		0.00	0.00, 0.01	0.210
Parent behavior	0.00	−0.04, 0.04	0.834	−0.03	−0.08, 0.03	0.317	0.02	−0.05, 0.08	0.612
**Sustained attention**									
Facial interest	0.01	−0.01, 0.04	0.396	0.03	0.01, 0.06	0.005*	−0.02	−0.07, 0.03	0.460
Duration of looking	0.19	−1.4, 1.8	0.818	0.64	−1.4, 2.7	0.527	−0.46	−3.4, 2.5	0.751
Gestures	−0.01	−0.02, 0.01	0.550	0.00	−0.03, 0.02	0.684	−0.02	−0.05, 0.02	0.379
Parent behavior	0.07	0.02, 0.11	**0.009**	0.08	−0.01, 0.16	0.070	0.06	−0.01, 0.13	0.071
Infant positive affect	−0.02	−0.06, 0.02	0.388	−0.03	−0.07, 0.02	0.200	−0.02	−0.10, 0.06	0.587
Infant negative affect	0.06	0.01, 0.11	**0.018**	0.06	−0.01, 0.14	0.098	0.06	−0.02, 0.14	0.114
Latency to look away	−0.12	−0.37, 0.13	0.330	−0.06	−0.46, 0.34	0.748	−0.19	−0.59, 0.20	0.317
**Maternal Separation**									
Facial fear	0.00	−0.09, 0.10	0.994	0.08	−0.12, 0.29	0.399	−0.04	−0.17, 0.09	0.547
Distress	−0.03	−0.17, 0.12	0.720	−0.01	−0.23, 0.21	0.913	−0.07	−0.32, 0.17	0.532
Latency to fear response	−0.68	−3.1, 1.8	0.575	−1.7	−5.5, 2.1	0.357	0.52	−3.4, 4.5	0.786
Bodily fear	0.03	−0.03, 0.09	0.346	0.05	−0.06, 0.16	0.365	0.02	−0.08, 0.11	0.709
Escape	0.00	−0.10, 0.09	0.917	0.07	−0.09, 0.23	0.354	−0.10	−0.23, 0.04	0.146

Controlled for Age at Assessment. Unpredictable mechanical toy task: n = 49 infants (21 male, 28 female); Sustained attention task: n = 52 infants (25 male, 27 female), except parent behavior (n = 51 infants, 24 male, 27 female), infant positive affect (n = 50 infants, 24 male, 26 female), and latency to look away (n = 51 infants, 24 male, 27 female); Maternal separation task: n = 51 infants (24 male, 27 female) except facial fear (n = 35 infants, 14 male, 21 female), and bodily fear (n = 45 infants, 19 male, 26 female). Bold values indicate *p* < 0.05; * indicates significance after correction for multiple testing (Benjamini-Hochberg). CI: Confidence Intervals.

**Fig. 1:**
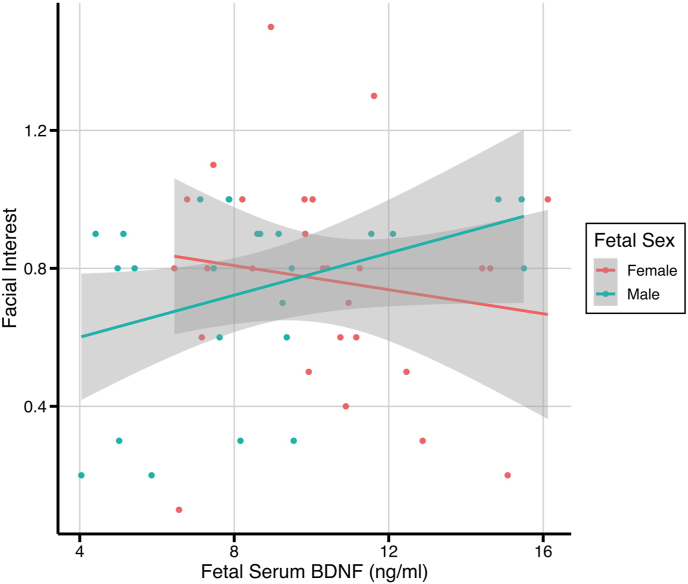
Cord blood serum BDNF levels plotted against facial interest scores from the sustained attention task, split by infant sex. Using a linear model to control for age at time of assessment, a 1 ng/ml increase in serum BDNF was associated with a 0.03 increase in facial interest scores in males only (95% confidence intervals: 0.01, 0.06; *p* = 0.005). 95% confidence intervals are shaded in gray.

The same parameters were assessed when split by infant sex. When female and male infants were analyzed separately, a sex-specific relationship was identified between intensity of facial interest and cord serum BDNF levels (Fig. 1; Table 3). For every 1 ng/ml increase in cord serum BDNF at birth, there was a 0.03 increase in facial interest scores, but only in male infants (*p* = 0.005). The associations identified in whole group analysis (parent behavior and infant negative affect) were not significant when analyzed by sex. When further controlling for parity, the relationship between facial interest scores and cord serum BDNF levels in males was maintained, and a further significant positive relationship between parent behavior and serum BDNF measurements in female infants was seen (*B* = 0.08, *p* = 0.038; Table S2), as previously identified in the whole group analysis. Only the relationship between facial interest in males and cord serum BDNF remained when correcting for multiple testing (correcting for 7 tests).

## 4 Discussion

In this study, we examined the relationship between serum BDNF levels in cord blood at birth and a number of infant outcomes at 12–14 months of age. Our key finding was the positive association between cord blood serum BDNF and intensity of facial interest in the sustained attention task, found only in the male infants (Fig. 1).

The sustained attention Lab-TAB task measures the duration of infants’ interest in a non-social manner. The ability to sustain attention develops early in life and goes on throughout childhood, and is critical to development of self-regulation (Brandes-Aitken et al., 2019). An inability to focus attention in infancy has been linked to ADHD-related behaviors later in childhood (Johnson et al., 2015). In this task, the intensity of facial interest scores positively correlated with serum BDNF levels at birth but only in male infants, a finding that remained significant even after correction for multiple testing. This is striking given the multiple lines of evidence linking BDNF and ADHD. The dopamine transporter knockout mouse, an animal model of the disorder, has altered expression of BDNF in the frontal cortex (Fumagalli et al., 2003); conversely animal models in which BDNF gene expression is altered show several phenotypes characteristic of ADHD, including hyperactivity (Kernie et al., 2000; Rios et al., 2001). Pharmacologically, BDNF levels have been shown to be altered in the prefrontal cortex in a rat model of ADHD in response to the ADHD medications, methylphenidate and atomoxetine (Fumagalli et al., 2010). Critically, methylphenidate has also been shown to increase blood levels of BDNF in children with ADHD (Akay et al., 2018; Amiri et al., 2013). This sex-specific association between cord serum BDNF and facial interest at 12–14 months could highlight a potential key consequence of the lower levels of serum BDNF seen in males at birth. With lower levels of the neurotrophin under healthy conditions, males may be more vulnerable to adverse effects mediated by reduced BDNF levels, e.g. as seen in premature infants (Cai et al., 2017; Cannon et al., 2008; Flock et al., 2016; Spulber et al., 2010). Several other parameters measuring attention in the sustained attention task showed no relationship with serum BDNF levels from birth, including duration of looking and latency to look away. Thus it is unclear whether attention capacity in male infants is holistically associated with serum BDNF levels from birth. Given the established relationship between ADHD and BDNF, further in-depth studies at later ages (when effortful control is more developed), are critical to help clarify this finding.

In the sustained attention task, serum BDNF levels were also associated with increased infant negative affect measures and increased parental interference, although these relationships did not remain after correcting for multiple testing. Infant negative affect includes fearfulness, irritability and frustration. Fear-based behaviors were also assessed in both the maternal separation and unpredictable mechanical toy tasks, with no further relationships identified. Parental behavior was also analyzed in the unpredictable mechanical toy task, however, no further relationship between parental behavior and cord serum BDNF measures was identified.

No significant associations were found between cord serum BDNF measurements and any of the behavioral parameters coded in the unpredictable mechanical toy or maternal separation tasks. These tasks primarily measure infant fear in response to either an unpredictable toy or to maternal separation. Infants who are more fearful or more reactive, showing behavioral inhibition, are reported to be more likely to develop anxiety symptoms later in childhood (Kagan, 2002); thus these results do not support serum BDNF values at birth having predictive value for later development of anxiety.

Assessing infant neurodevelopment, we found no associations between birth BDNF levels and BSID-III cognitive or language outcomes at this age. This study is the first to our knowledge examining the relationship between cord serum BDNF levels at birth with BSID-III scores at 12–14 months. One previous study assessed the relationship between BSID-III scores and cord serum BDNF at 24 months and reported no association between serum BDNF levels at birth and BSID-III cognitive, language or motor scores (Simpson et al., 2019). Perera et al. (2015) identified a positive association between plasma BDNF from cord blood and BSID-III mental development index scores at 2 years; however this relationship disappeared by 3 years of age (Perera et al., 2015). A lack of association between plasma BDNF at birth and BSID-III scores between 15 and 30 months has also been reported (Dietrick et al., 2020). Furthermore, Su et al. (2021) identified a positive correlation between BDNF levels and language scores when both measures were assessed at 6 months and 12 months, but this was only present in infants from mothers with gestational diabetes (Su et al., 2021), and the relationship between birth BDNF levels and Year 1 outcomes was not directly assessed. Two studies have used the Gesell Developmental Schedule to assess infant outcomes at 12 months of age. Both reported that BDNF levels in cord serum were positively associated with social domain development quotients (Wang et al., 2016; Yu et al., 2016); one found that serum BDNF levels were also negatively associated with fine motor scores (Yu et al., 2016), a domain not measured in the current study. The difference between plasma and serum BDNF levels in all populations studied, and differences between the Gesell Developmental Schedule and the BSID-III, can easily account for the differences between these studies and the findings reported here.

### 4.1 Strengths

One of the key strengths of the study was the relative homogeneity of the cohort. The Grown in Wales Study is a cohort of women all living in the South Wales area. Further, as all women in the cohort underwent an ELCS, cord blood collection could be performed under relatively stable conditions for each participant. A further significant advantage of this study is the use of researcher-observed tasks to assess infant behavior and neurodevelopment; these allow independent assessment of infants in a standardized environment.

### 4.2 Limitations

The participants in this study all underwent an ELCS. It has previously been reported that significant differences in infant serum BDNF measures are associated with different modes of delivery (with measurements from ELCS lower than vaginal deliveries) (Flock et al., 2016), thus a wider assessment across different modes of delivery would be important for future studies. In addition, data analyzed here were derived only from white individuals, which may limit the applicability to the wider community. The Grown in Wales cohort was also recruited from a relatively limited geographical area in South Wales; future research should replicate these findings in more diverse populations.

## 5 Conclusion

To our knowledge, this is the earliest timepoint at which BDNF levels have been associated with a behavior in human infants (Supplementary Table 1**)**. The association between higher BDNF levels and higher intensity of facial interest would be consistent with the proposal that lower BDNF levels increase the risk of poorer attention in males.

## Data availability

Data not available; data that has been used is confidential.

## Author contributions

Hayley Dingsdale: Conceptualization, Data Curation, Formal analysis, Investigation, Methodology, Resources, Writing - original draft, Writing - review & editing; Samantha M Garay: Data Curation, Methodology, Writing - review & editing; Hannah R Tyson: Data Curation, Writing - review & editing; Katrina A Savory: Data Curation, Writing - review & editing; Lorna A Sumption: Data Curation, Writing - review & editing; Jemima S Kelleher: Data Curation, Writing - review & editing; Kate Langley: Writing - review & editing; Stephanie Van Goozen: Funding acquisition, Writing - review & editing; Rosalind M John: Conceptualization, Funding acquisition, Project administration, Resources, Supervision, Writing - original draft, Writing - review & editing.

## Funding

Funding is gratefully acknowledged from 10.13039/100012107The Waterloo Foundation (HD: Child Development Fund Research Grant No. 1403-4535; KAS: 918–3022), from the 10.13039/501100000265MRC (SMG: GW4 BioMed PhD Studentship MR/N013794/1), from the 10.13039/501100000268BBSRC (LAS: SWBio PhD Studentship BB/M009122/1), and from the 10.13039/100010269Wellcome Trust (HRT: 10.13039/100010269Wellcome Trust doctoral training grant 19100-BV19108003). The Grown in Wales Study was funded by 10.13039/501100000265MRC grant MR/M013960/1. Funding sources had no role in the study design; in the collection, analysis and interpretation of data; in the writing of the report; nor in the decision to submit the article for publication.

## Declarations of interest

None.
