# Supplementary material for: Cord serum brain-derived neurotrophic factor levels at birth associate with temperament outcomes at one year
Source: J Psychiatr Res. 2022 Jun;150:47–53. doi: 10.1016/j.jpsychires.2022.03.009 (PMC9225956; doi:10.1016/j.jpsychires.2022.03.009)
Supplement: Multimedia component 1 [file mmc1.docx]

**Supplemental Tables**

**Supplementary Table 1:** **Previous literature reporting on ADHD and blood BDNF measurements.** *Search terms: “BDNF” and “ADHD” or “attention deficit” or “attention hyperactivity” and studies found within relevant papers. Studies were only included if they measured either plasma or serum levels of BDNF in the context of ADHD. Only studies with a manuscript available in English were included. Studies are ordered by year, then alphabetically by first author.* Abbreviations: ADHD: Attention deficit-hyperactivity disorder; ADS: Computerized ADHD diagnostic system; ARS: ADHD rating scale; BDNF: Brain-derived neurotrophic factor; CBCL: Child Behavior Checklist; CCPT: Computerized continuous performance test; CCTT: Children’s color trails test; DSM: Diagnostic and Statistical Manual of Mental Disorders; HC: Healthy control*;* IQR: Interquartile range; K-SADS-PL: Kiddie-Schedule for Affective Disorders and Schizophrenia for School-Age Children – Present and the Lifetime; KEDI-WISC: Korean Educational Development Institute-WISC; OR: Odds ratio; PAD: Predominant attention deficits; PHI/CD: Predominant hyperactive-impulsive subtype with co-occurring conduct disorder; RT: Room temperature; SCID: Structured Clinical Interview for DSM-IV; SD: Standard deviation; SE: Standard error; SED: Socioeconomic disadvantage; WISC: Wechsler Intelligence Scale for Children*.*

| **Paper** | **Sample Type & No.** | **Ages (years)** | **Assessments** | **Sample**  **Preparation** | **Average BDNF (ng/ml)** | **Findings Relevant to Current Study** |
| --- | --- | --- | --- | --- | --- | --- |
| **Shim *et al.,* 2008 (1)** | Plasma from 41 drug-naïve children with ADHD (78% male), and 107 healthy controls (38% male). | Mean (SD):  HC: 9.0 (1.3).  ADHD: 8.8 (2.3). | K-SADS-PL, Korean version;  KEDI-WISC for intelligence; Korean-ARS; computerized ADHD diagnostic system. | 10 ml blood from the antecubital vein collected between 0800-1200, and placed in a lithium heparin vacuum tube. | Mean (SD):  HC: 0.576 (0.032).  ADHD: 0.841 (0.054). | Plasma BDNF levels were higher in patients with ADHD than HC (*p*<0.001), which remained when adjusted for age and gender.  A significant correlation between plasma BDNF and omission error in the ADS was seen in patients with ADHD (Pearson’s *r* = 0.345, *p*<0.05). |
| **Amiri *et al.,* 2013 (2)** | Plasma from 28 children with ADHD (86% male) before and after 6 weeks treatment with methylphenidate. | Mean (SD):  7.6 (2). | K-SADS-PL, Persian version; WISC, or Wechsler Preschool and Primary Scale of Intelligence; severity by Conner’s Parents Rating Scale. | 5 ml blood from the antecubital vein was collected into heparin vacuum tubes between 0800-1200. | Mean (SD):  Before treatment: 0.193 (0.095).  After treatment: 0.271 (0.111). | BDNF plasma levels were significantly increased post-treatment (*p*=0.002). There was also a significant negative correlation between BDNF plasma levels prior to treatment and the post-treatment improvement in hyperactivity (Pearson’s correlation = -0.395, *p*=0.037). |
| **Corominas-Roso *et al.,* 2013 (3)** | Serum from 54 adults with ADHD (69% male), and 59 unrelated, healthy controls (64% male). | Mean (SD):  HC: 34.1 (9.40).  ADHD: 33.4 (8.99). | Conners Adult ADHD Diagnostic Interview and Conners’ Adult ADHD Rating; Wender Utah Rating Scale used for retrospective symptomatology. | 8 ml blood from the antecubital vein collected into anticoagulant-free tubes between 1000-1200. Incubation in the refrigerator for 2 h, before centrifugation at 2465 x *g* for 10 min at 4 °C, and storage at -80 °C. | Mean (SD):  HC: 69.77 (23.20).  ADHD: 52.13 (20.76). | Serum BDNF levels were lower in adults with ADHD than HC (*p*<0.0001).  No correlation between serum BDNF levels and scores on the Conners’ Adult ADHD Rating. |
| **Li *et al.,* 2014 (4)** | Plasma from 170 children with ADHD (medication-naïve; 51% male) and 155 unaffected controls (49% male). | Mean (unclear if SE/SD):  HC: 9.5 (1.4).  ADHD: 9.4 (2.3). | Clinical diagnosis confirmed by Clinical Diagnostic Interview Scale; severity assessed with the ADHD Diagnostic Scale Parent Version. | Blood from the antecubital vein collected into lithium-heparin tubes between 1000-1200. After max. 30 min, transferred to small Eppendorf tubes, and centrifuged at 10,000 x *g* at 0 °C, before storage of plasma at -80 °C. | Mean (unclear if SE/SD):  HC: 0.184 (0.209).  ADHD: 0.257 (0.186). | Mean plasma BDNF levels were higher in patients with ADHD than HC (*p*=0.001) after adjusted for age and sex. This difference was also present when analysis was stratified by sex. |
| **Ramos-Quiroga *et al.,* 2014 (5)** | Serum from 54 adults with ADHD (69% male), before and after 3 month treatment with atomoxetine (35 completed study). | Mean (unclear if SE/SD):  33.4 (8.99). | Clinical diagnosis confirmed by SCID I and II; Spanish version of Conners Adult ADHD Diagnostic Interview for DSM-IV and Conners’ Adult ADHD Rating Scale; Wender Utah Rating Scale for retrospective symptomatology. | 8 ml blood from the antecubital vein collected into anticoagulant-free tubes between 1000-1200. Incubation at 4 °C for 2 h, before centrifugation at 3500 for 10 min at 4 °C. | Mean (SD):  Pre-treatment: 52.13 (20.76).  Post-treatment: 47.38 (14.92). | No significant difference in serum BDNF was seen post-treatment. However, when splitting by ADHD subtype, the inattentive subgroup showed a decrease of serum BDNF post-treatment (*p*=0.05) that was not present in the combined subtype or whole patient group. Serum BDNF at baseline correlated significantly with improvement of inattention subscale of the Connors’ Adult ADHD Rating Scale (*r*=0.354; *p*=0.034). |
| **Sahin *et al.,* 2014 (6)** | Blood samples (not stated if BDNF measured in plasma or serum) at baseline and at 2 months from 30 children with ADHD undergoing methylphenidate treatment (80% male) and 20 unaffected controls (90% male). | Mean (SD):  HC: 9.7 (2.29).  ADHD: 9.5 (2.83). | K-SADS-PL, Turkish version; the ADHD group also completed the Clinical Global Impression Scale. | Blood collected after 12 h overnight fasting. Serum and plasma separated and stored at -80 °C. | Mean (SD):  HC: 317.2 (100.6).  ADHD pre-treatment: 341.8 (86.4).  ADHD post-treatment: 300.8 (128.1). | No baseline difference in BDNF levels between HC and the patients with ADHD. In patients undergoing treatment with methylphenidate, BDNF levels were significantly reduced after 2 months (*p*=0.048). |
| **Scassellati *et al.,* 2014 (7)** | Serum from 45 drug-naïve children with ADHD (93% male), and 45 unrelated, healthy controls (91% male). | Mean (SD):  HC: 10.3 (2.04).  ADHD: 10.7 (2.48). | Conner’s Rating Scale Revised; ADHD diagnosis according to the DSM-IV and the guidelines of the Italian Institute of Health; revised Touwen neurological tests were performed. | Venous blood collected into anticoagulant-free tubes between 0800-0900 after an overnight fast. Incubated at RT for 2 h, then 4 °C for 1 h before centrifugation at 1620 x *g* for 15 min. Storage at -80 °C. | Mean (SD):  HC: 38.82 (8.29).  ADHD: 39.33 (10.41). | BDNF serum levels were not significantly different in patients with ADHD (*p*=0.80). |
| **Lee *et al.,* 2015 (8)** | Serum at baseline and at 32 weeks from 39 children with ADHD – 20 undergoing hippotherapy (75% male) and 19 in the control group (53% male). | Mean (SD):  Control group: 12.3 (0.56).  Hippotherapy group: 11.6 (1.85). | Kiddie-Schedule for Affective Disorders and Schizophrenia. | Blood taken from the forearm vein, centrifuged at 1000 x *g* for 15 min. | Mean (SD)^a^:  Baseline:  Control: 13.65 (5.66).  Hippotherapy group: 13.30 (3.96).  Change after 32 weeks:  Control: 0.211 (6.86).  Hippotherapy group: 1.66 (2.78). | No significant effect of hippotherapy on serum BDNF levels in children with ADHD. |
| **Mansur *et al.,* 2016 (9)** | Serum from 495 children (55% male). | Mean (SD):  10.1 (1.88). | CBCL. | Blood collected between 1000-1600 and allowed to clot at RT. Centrifuged between 1,000-2,000 x *g* for 10 min in a refrigerated centrifuge, before storage at -80 °C. | Median (IQR):  0.0260 (0.0201-0.0335). | BDNF serum levels were higher in female children (*p*=0.002). There was a positive correlation between SED and serum BDNF (*r*=0.098, *p*=0.022), but this disappeared when controlling for age, gender, and ethnicity (*p*=0.077). BDNF was associated with anxiety, attention and conduct scales of the CBCL when analyzed separately, i.e. in a model not including SED. |
| **Şimşek *et al.,* 2016 (10)** | Serum from 49 children with ADHD (treatment-naïve; 86% male), and 40 healthy children (80% male). | Mean (unclear if SE/SD):  HC: 8.7 (2.2).  ADHD: 8.6 (2.4). | K-SADS-PL-Turkish version; Turgay DSM-IV-based Child and Adolescent Behavior Disorders Screening and Rating Scale; Stroop Test (TBAG Form). | 2 ml blood collected between 0900-1200 into gel tubes. Stored at RT for 15 min to facilitate clotting and centrifuged at 5000 rpm for 6 min. Serum separated and stored at -80 °C. | Average (type unclear, however the data is stated as not being normally distributed):  HC: 8.7 (12.3).  ADHD: 11.1 (16.8). | No significant differences in serum BDNF levels between children with ADHD and HC. BDNF levels also did not differ between ADHD subtypes, nor was there any relationship between ADHD symptom severity and BDNF levels. No significant relationship between Stroop test interference effect scores and BDNF levels. |
| **Yeom *et al.,* 2016 (11)** | Serum and plasma from 28 pre-school children (46% male). | Mean (SD):  6.2 (0.60). | KEDI-WISC for intelligence; CCPT as diagnostic tool of ADHD; CCTT and Stroop color-word test; Korean parent-report version of the CBCL; ADHD screening and symptom severity by ARS. | Blood drawn at 1400. For serum, blood was allowed to clot for 30 min in serum separator tubes, and centrifuged for 15 min at ~ 1000 x *g* before separation and storage at 80 °C. Plasma collected on ice using EDTA tubes, and centrifuged for 15 min at 1000 x *g* at 4 °C. Separated plasma then recentrifuged at 10,000 x *g* for 10 min at 4 °C, before separation and storage at -80 °C. | Mean (SD):  Serum – ages 5-6: 22.87 (8.25); ages 6-7 22.59 (8.58). Plasma – ages 5-6: 2.687 (1.77); ages 6-7: 3.00 (1.61). | No correlation of serum or plasma BDNF levels with CCPT, ARS, CCTT, or Stroop color-word test. Total behavioral and attention problem sections of the CBCL were positively related to plasma levels of BDNF (*r*=0.41, *p*=0.03; *r*=0.44, *p*=0.02 respectively). |
| **Zeni *et al.,* 2016 (12)** | Serum from 34 patients with bipolar disorder with ADHD (68% male), and 27 patients with ADHD only (82% male). | Mean (SD):  Bipolar & ADHD: 11.5 (2.81).  ADHD only: 10.6 (3.34). | K-SADS-PL; diagnosis of bipolar disorder and ADHD by DSM-IV criteria. | 8 ml blood withdrawn from each subject. | Mean (SD) pg BDNF/µg protein^c^:  Bipolar & ADHD: 0.69 (0.3).  ADHD: 0.48 (0.15). | Serum BDNF was higher in patients with bipolar disorder co-occurring with ADHD than in patients with ADHD only (*p=*0.005). |
| **Allred *et al.,* 2017 (13)** | Blood spots from 793 infants (born before 28 weeks gestation) over the 1^st^ month after birth, followed up by assessment at 10 years of age. | Average not given, blood taken during 1^st^ month; assessment at 10 years of age. | Parent/caregiver completed Child Symptom Inventory-4 Parent Checklist; current teacher asked to complete Child Symptom Inventory-4 Teacher Checklist. | Blood drops collected on filter paper on postnatal days 1 (range 1-3 days), 7 (range 5-8 days), 14 (range 12-15 days), 21 (range 19-23 days) and 28 (range 26-29 days).  Dried blood stored at -70 °C with desiccant. | Not stated. | Top-quartile BDNF levels on postnatal day 7 were associated with increased OR of ADHD as defined by their teacher (OR: 1.8; 95% CI: 1.1, 2.8). Top-quartile BDNF was also associated with a modulatory role: when IL-8 measurements were in the top-quartile, BDNF measurements also in the top-quartile were associated with a reduced risk of teacher-identified ADHD symptoms. However, this was only one instance among nine opportunities. Allred *et al*. conclude that their paper adds support to “no-association” between ADHD and BDNF in this context. |
| **Bilgiç *et al.,* 2017 (14)** | Serum from 110 treatment-naïve children with ADHD (80% male), and 44 healthy children (70% male). | ^b^Mean (unclear if SE/SD):  HC: 10.9 (2.8).  ADHD: 10.3 (2.1). | K-SADS-PL; diagnosis of combined ADHD according to DSM 5; Conners’ Parent Rating Scale-Revised Short and Conners’ Teacher Rating Scale-Revised Short. | Blood collected from antecubital vein between 0800-1000 after an overnight fast. Samples centrifuged before storage at -80 °C. | Mean (unclear if SE/SD):  HC: 16.4 (7.6).  ADHD: 14.7 (8.4). | BDNF serum levels were not significantly different in children with ADHD than HC. There were also no sex-dependent differences in serum BDNF levels. |
| **Cubero-Millán *et al.,* 2017 (15)** | Serum from 107 children with ADHD (treatment-naïve; 79% male; before and after ~4.6 months methylphenidate treatment), and 41 healthy children (73% male). | Mean (SD):  HC: 10.2 (2.58).  ADHD-PAD: 9.6 (2.77).  ADHD-PHI/CD: 9.3 (2.41). | DSM-IV-TR criteria assessment; an EDAH scale (Spanish acronym of evaluation of deficit of attention and hyperactivity scale), with one completed by the child’s parents, and one completed by the teacher; d2 Test (measure of attention). | Blood collected at 2000 and 0900. Serum separated and stored at -30 °C. | Mean (SD):  Baseline:  HC: 34.39 (11.88).  ADHD: 30.16 (12.63).  Post-treatment:  ADHD: 28.05 (12.20). | At baseline, children with ADHD had significantly lower serum BDNF levels than HC (*p*=0.028). Subtype-specific, there were significantly lower serum BDNF levels in the ADHD-PHI/CD subgroup (29.91 ± 12.57; *p*=0.024) compared to HC. There was also a significant day/night difference in the ADHD-PAD subgroup (*p*=0.0019) at baseline that was no longer present post-treatment with methylphenidate (which significantly reduced the morning serum BDNF values; *p*=0.021). |
| **Lee *et al.,* 2017 (16)** | Serum from 16 children with ADHD, before and after hippotherapy (once or twice/week). Seven of these children attended a further hippotherapy program with neurofeedback. | Mean (SD):  1/week group: 11.8 (1.28).  2/week group: 12.0 (1.51).  Combined: 11.4 (0.98). | Psychological medical examination conducted for calculating the attention quotient for selecting patients. Comprehensive attention test used in the secondary training. | Blood collected from the forearm venous vein and stored in serum tubes. Centrifuged at 1000 x *g* for 15 min. | Mean (SD):  Baseline:  1/week: 1.77 (0.36).  2/week: 1.97 (0.43).  Combined: 1.44 (0.37).  Post-treatment  1/week: 1.63 (0.28).  2/week: 1.98 (0.43).  Combined: 1.53 (0.35). | There was a significant difference in the change seen post-training between the once/week (decreased) and twice/week (increased) groups (*p*<0.05). |
| **Vogel *et al.,* 2017 (17)** | Serum from 2307 adults as part of the Netherlands Study of Depression and Anxiety (34% male). 7.9% had High ADHD symptoms from this cohort. | Mean (SD):  42.2 (13.1). | Conners’ Adult ADHD Rating Scale – Screening Version (self-report). *T*-scores of 65 or higher were above the clinical relevant cut-off and defined as ‘High ADHD symptoms’. | Serum separated immediately after draw and stored at -85 °C.  Further described in (18): blood collected before 1000 after an overnight fast. | Mean (SD): 9.09 (3.30). | No significant associations found between any ADHD symptomatology and serum BDNF levels. |
| **Akay *et al.,* 2018 (19)** | Serum from 50 children with ADHD (treatment-naïve; 100% male; before and after 8 weeks methylphenidate treatment), and 50 healthy children (100% male). | Mean (SD):  HC: 8.8 (1.1).  ADHD: 8.8 (1.5). | ADHD diagnosis in accordance with DSM-IV-R, using K-SADS-PL-Turkish version; healthy controls: parents completed a clinical interview, K-SADS-PL and CBCL. ADHD symptom severity was assessed by physicians applying the Du Paul ADHD Rating Scale IV and Clinical Global Impression-severity. | 10 ml blood collected between 0900-1000, after at least 12 h fasting. Collected into tubes without anticoagulant and incubated at RT for 30 min. Centrifuged at 3000 x *g* for 10 min and stored at -85 °C. | Mean (SD):  Baseline:  HC: 2.99 (1.42).  ADHD-Total: 2.63 (1.53).  Post-treatment  ADHD-Total: 3.26 (1.91). | No baseline difference between treatment-naïve patients with ADHD and HC. Comparing subtypes, serum BDNF levels were significantly lower in patients with PAD (*p*=0.02), with no difference between the combined or PHI subtypes. Methylphenidate treatment for 8 weeks induced a significant increase in serum BDNF (*p*=0.04), which was significantly greater in patients with PAD (*p*=0.005). |
| **Seyedi *et al.,* 2019 (20)** | Serum from 86 children with ADHD, divided into 12 weeks placebo or 12 weeks treatment with Vitamin D3 supplementation. | *Full text unavailable.* | *Full text unavailable.* | *Full text unavailable.* | *Full text unavailable.* | Vitamin D3 supplementation did not significantly alter serum BDNF levels. |
| **Skogstrand *et al.,* 2019 (21)** | Dried blood spots from 751 children with ADHD (73% male), and 2423 children without ADHD (52% male). | Blood taken at 5-7 days of age. Average age of ADHD diagnosis (median (IQR)): 10 (8-15). | Samples selected from the iPSYCH cohort. | Dried blood spots taken between 5-7 days of birth. | Values given in boxplots only. | BDNF levels in dried blood spots were not associated with increased odds of developing ADHD. |
| **Wang *et al.,* 2019 (22)** | Plasma from 136 children with ADHD (treatment-naïve; 79% male), and 71 healthy children (63% male). | Mean (SD):  Male HC: 9.4 (2.4).  Male ADHD: 9.0 (2.3).  Female HC: 9.9 (2.6).  Female ADHD: 8.0 (1.3). | ADHD diagnosis in accordance with DSM-IV-TR and K-SADS-E; The Swanson, Nolan, and Pelham Version IV Scale parent form, and teacher form, were completed. The Conners’ continuous performance test. | Blood collected at 0800 after overnight fasting. | Mean (SD):  Male HC: 3.17 (3.84).  Male ADHD: 4.57 (4.43).  Female HC: 4.69 (4.36).  Female ADHD: 3.01 (2.99). | Plasma levels of BDNF did not differ significantly between patients with ADHD and HC. However, split by sex, males with ADHD had higher BDNF levels than HC (*p*=0.027), and females with ADHD had lower BDNF levels than HC (*p*=0.014). In girls, there was a negative correlation between plasma BDNF levels and both parent-rated inattention symptoms and omission scores in the CPT (*r=*-0.322, *p*=0.019; *r*=-0.356, *p*=0.008, respectively). Findings not maintained after correcting for multiple testing. |
| **Yurteri *et al.,* 2019 (23)** | Serum from 49 children with ADHD (treatment-naïve; 69% male), and 36 healthy children (67% male). | Median (IQR):  HC: 10.5 (1.7).  ADHD: 10.3 (2.4). | ADHD diagnosis in accordance with DSM-V and K-SADS-PL-Turkish version; ADHD symptom severity was assessed by the Du Paul ADHD Rating Scale, and Strengths and Difficulties Questionnaire-Parent version. | Venous blood collected between 0800-1000, after 12 h fasting. Blood samples were centrifuged and stored at -80 °C. | Median (IQR):  HC: 14.87 (7.22).  ADHD: 13.41 (9.36). | No difference in serum BDNF levels between patients with ADHD and HC, or when ADHD subtypes were compared. No correlation between ADHD symptoms and serum BDNF levels. |
| **Chang *et al.,* 2020 (24)** | Plasma from 98 youths with ADHD (treatment-naïve or 6 month treatment-free; 86% male), and 21 youths without ADHD (71% male). | Mean (SD):  HC: 9.2 (2.96).  ADHD: 9.3 (3.05). | ADHD diagnosis in accordance with DSM-V-TR; Severity by the Chinese version of the Swanson, Nolan, and Pelham Version IV. | Venous blood collected into EDTA tubes, between 0800-1000, after 12 h fasting. Centrifugation at 1200 x *g* for 10 min (at 25 °C), before storage at -80 °C. | Mean (SD):  HC: 1.22 (0.456).  ADHD: 0.779 (0.381). | Plasma BDNF levels were lower in the patients with ADHD than in HC (*p*<0.0001). |

^a^Results were originally presented as pg BDNF, derived from measurement of 100 µl serum; these have been converted to ng/ml.

^b^Although not explicitly stated, group age averages were compared by Student’s *t* test, indicating that this data is likely normal and the mean is therefore presented.

^c^BDNF values presented as pg/µg protein instead of ng/ml.

1. S. H. Shim *et al.*, Increased levels of plasma brain-derived neurotrophic factor (BDNF) in children with attention deficit-hyperactivity disorder (ADHD). *Prog Neuropsychopharmacol Biol Psychiatry* **32**, 1824-1828 (2008).

2. A. Amiri *et al.*, Changes in plasma Brain-derived neurotrophic factor (BDNF) levels induced by methylphenidate in children with Attention deficit-hyperactivity disorder (ADHD). *Prog Neuropsychopharmacol Biol Psychiatry* **47**, 20-24 (2013).

3. M. Corominas-Roso *et al.*, Decreased serum levels of brain-derived neurotrophic factor in adults with attention-deficit hyperactivity disorder. *Int J Neuropsychopharmacol* **16**, 1267-1275 (2013).

4. H. Li *et al.*, Sex-specific association of brain-derived neurotrophic factor (BDNF) Val66Met polymorphism and plasma BDNF with attention-deficit/hyperactivity disorder in a drug-naive Han Chinese sample. *Psychiatry Res* **217**, 191-197 (2014).

5. J. A. Ramos-Quiroga *et al.*, Changes in the serum levels of brain-derived neurotrophic factor in adults with attention deficit hyperactivity disorder after treatment with atomoxetine. *Psychopharmacology (Berl)* **231**, 1389-1395 (2014).

6. S. Sahin *et al.*, Effect of methylphenidate treatment on appetite and levels of leptin, ghrelin, adiponectin, and brain-derived neurotrophic factor in children and adolescents with attention deficit and hyperactivity disorder. *Int J Psychiatry Clin Pract* **18**, 280-287 (2014).

7. C. Scassellati *et al.*, Serum brain-derived neurotrophic factor (BDNF) levels in attention deficit-hyperactivity disorder (ADHD). *Eur Child Adolesc Psychiatry* **23**, 173-177 (2014).

8. N. Lee, S. Park, J. Kim, Effects of hippotherapy on brain function, BDNF level, and physical fitness in children with ADHD. *J Exerc Nutrition Biochem* **19**, 115-121 (2015).

9. R. B. Mansur *et al.*, Socioeconomic Disadvantage Moderates the Association between Peripheral Biomarkers and Childhood Psychopathology. *PLoS One* **11**, e0160455 (2016).

10. S. Simsek *et al.*, Evaluation of the Relationship between Brain-Derived Neurotropic Factor Levels and the Stroop Interference Effect in Children with Attention-Deficit Hyperactivity Disorder. *Noro Psikiyatr Ars* **53**, 348-352 (2016).

11. C. W. Yeom, Y. J. Park, S. W. Choi, S. Y. Bhang, Association of peripheral BDNF level with cognition, attention and behavior in preschool children. *Child Adolesc Psychiatry Ment Health* **10**, 10 (2016).

12. C. P. Zeni *et al.*, BDNF Val66Met polymorphism and peripheral protein levels in pediatric bipolar disorder and attention-deficit/hyperactivity disorder. *Acta Psychiatr Scand* **134**, 268-274 (2016).

13. E. N. Allred *et al.*, Systemic Inflammation during the First Postnatal Month and the Risk of Attention Deficit Hyperactivity Disorder Characteristics among 10 year-old Children Born Extremely Preterm. *J Neuroimmune Pharmacol* **12**, 531-543 (2017).

14. A. Bilgic, A. Toker, U. Isik, I. Kilinc, Serum brain-derived neurotrophic factor, glial-derived neurotrophic factor, nerve growth factor, and neurotrophin-3 levels in children with attention-deficit/hyperactivity disorder. *Eur Child Adolesc Psychiatry* **26**, 355-363 (2017).

15. I. Cubero-Millan *et al.*, BDNF concentrations and daily fluctuations differ among ADHD children and respond differently to methylphenidate with no relationship with depressive symptomatology. *Psychopharmacology (Berl)* **234**, 267-279 (2017).

16. N. Lee, S. Park, J. Kim, Hippotherapy and neurofeedback training effect on the brain function and serum brain-derived neurotrophic factor level changes in children with attention-deficit or/and hyperactivity disorder. *J Exerc Nutrition Biochem* **21**, 35-42 (2017).

17. S. W. N. Vogel *et al.*, Attention-deficit/hyperactivity disorder symptoms and stress-related biomarkers. *Psychoneuroendocrinology* **79**, 31-39 (2017).

18. M.L. Molendijk *et al.,* Gender specific associations of serum levels of brain-derived neurotrophic factor in anxiety. *World J Biol Psychiatry* **13**, 535-543 (2012).

19. A. P. Akay *et al.*, Serum brain-derived neurotrophic factor levels in treatment-naive boys with attention-deficit/hyperactivity disorder treated with methylphenidate: an 8-week, observational pretest-posttest study. *Eur Child Adolesc Psychiatry* **27**, 127-135 (2018).

20. M. Seyedi *et al.*, The Effect of Vitamin D3 Supplementation on Serum BDNF, Dopamine, and Serotonin in Children with Attention-Deficit/Hyperactivity Disorder. *CNS Neurol Disord Drug Targets* **18**, 496-501 (2019).

21. K. Skogstrand *et al.*, Reduced neonatal brain-derived neurotrophic factor is associated with autism spectrum disorders. *Transl Psychiatry* **9**, 252 (2019).

22. L. J. Wang *et al.*, Peripheral Brain-Derived Neurotrophic Factor and Contactin-1 Levels in Patients with Attention-Deficit/Hyperactivity Disorder. *J Clin Med* **8**, 1366 (2019).

23. N. Yurteri, I. E. Sahin, A. E. Tufan, Altered serum levels of vascular endothelial growth factor and glial-derived neurotrophic factor but not fibroblast growth factor-2 in treatment-naive children with attention deficit/hyperactivity disorder. *Nord J Psychiatry* **73**, 302-307 (2019).

24. J. P. Chang *et al.*, Cortisol, inflammatory biomarkers and neurotrophins in children and adolescents with attention deficit hyperactivity disorder (ADHD) in Taiwan. *Brain Behav Immun* **88**, 105-113 (2020).

**Table S2. Relationship between infant serum BDNF levels at birth and infant behavior in three Lab-TAB tasks after additionally controlling for parity**

|  | **All** | | | **Male** | | | **Female** | | |
| --- | --- | --- | --- | --- | --- | --- | --- | --- | --- |
|  | *B* | 95% CI | *p* | *B* | 95% CI | *p* | *B* | 95% CI | *p* |
| **Unpredictable mechanical toy (fear)** |  |  |  |  |  |  |  |  |  |
| Facial fear | -0.06 | -0.16, 0.04 | 0.213 | -0.04 | -0.20, 0.12 | 0.585 | -0.11 | -0.27, 0.05 | 0.172 |
| Distress | -0.08 | -0.20, 0.04 | 0.187 | -0.09 | -0.24, 0.05 | 0.196 | -0.13 | -0.34, 0.08 | 0.219 |
| Bodily fear | 0.00 | -0.04, 0.04 | 0.984 | 0.01 | -0.07, 0.09 | 0.784 | 0.00 | -0.06, 0.05 | 0.870 |
| Intensity of escape | -0.01 | -0.05, 0.04 | 0.753 | 0.01 | -0.04, 0.07 | 0.567 | -0.03 | -0.11, 0.05 | 0.395 |
| Startle response | 0.00 | 0.00, 0.00 | 0.191 | 0.00 | 0.00, 0.00 |  | 0.00 | 0.00, 0.01 | 0.484 |
| Parent behavior | 0.00 | -0.05, 0.04 | 0.847 | -0.03 | -0.09, 0.03 | 0.272 | 0.01 | -0.06, 0.08 | 0.701 |
| **Sustained attention** |  |  |  |  |  |  |  |  |  |
| Facial interest | 0.01 | -0.01, 0.04 | 0.362 | 0.03 | 0.01, 0.06 | **0.005** | -0.02 | -0.07, 0.04 | 0.552 |
| Duration of looking | 0.20 | -1.4, 1.8 | 0.806 | 0.93 | -1.1, 2.9 | 0.345 | 0.05 | -3.1, 3.2 | 0.975 |
| Gestures | 0.00 | -0.02, 0.01 | 0.597 | -0.01 | -0.03, 0.02 | 0.624 | -0.01 | -0.05, 0.02 | 0.487 |
| Parent behavior | 0.07 | 0.02, 0.12 | **0.007** | 0.07 | -0.01, 0.16 | 0.093 | 0.08 | 0.00, 0.15 | **0.038** |
| Infant positive affect | -0.02 | -0.06, 0.02 | 0.395 | -0.03 | -0.08, 0.02 | 0.209 | -0.01 | -0.09, 0.07 | 0.722 |
| Infant negative affect | 0.06 | 0.01, 0.11 | **0.019** | 0.06 | -0.02, 0.14 | 0.130 | 0.06 | -0.03, 0.14 | 0.174 |
| Latency to look away | -0.14 | -0.39, 0.12 | 0.279 | -0.01 | -0.40, 0.38 | 0.950 | -0.20 | -0.62, 0.23 | 0.345 |
| **Maternal Separation** |  |  |  |  |  |  |  |  |  |
| Facial fear | 0.01 | -0.09, 0.11 | 0.840 | 0.07 | -0.17, 0.30 | 0.548 | -0.02 | -0.16, 0.12 | 0.738 |
| Distress | -0.02 | -0.17, 0.13 | 0.750 | -0.01 | -0.24, 0.22 | 0.918 | -0.06 | -0.32, 0.20 | 0.624 |
| Latency to fear response | -0.68 | -3.2, 1.8 | 0.588 | -1.7 | -5.6, 2.2 | 0.382 | 0.80 | -3.3, 5.0 | 0.693 |
| Bodily fear | 0.03 | -0.04, 0.09 | 0.424 | 0.06 | -0.05, 0.17 | 0.296 | 0.02 | -0.08, 0.12 | 0.724 |
| Escape | -0.01 | -0.10, 0.09 | 0.910 | 0.07 | -0.09, 0.24 | 0.364 | -0.11 | -0.25, 0.03 | 0.128 |

Controlled for age at assessment and parity. Unpredictable mechanical toy task: n=49 infants (21 male, 28 female); Sustained attention task: n=52 infants (25 male, 27 female), except parent behavior (n=51 infants, 24 male, 27 female), infant positive affect (n=50 infants, 24 male, 26 female), and latency to look away (n=51 infants, 24 male, 27 female); Maternal separation task: n=51 infants (24 male, 27 female), except facial fear (n=35 infants, 14 male, 21 female), and bodily fear (n=45 infants, 19 male, 26 female). Bold values indicate *p* < 0.05. CI: Confidence Intervals.
